# Synthesis, characterization and evaluation of antioxidant activities of some novel chalcones analogues

**DOI:** 10.1186/1752-153X-8-32

**Published:** 2014-05-07

**Authors:** Siham Abdelrahmane Lahsasni, Faeza Hamad Al Korbi, Nabilah Abdel-Aziz Aljaber

**Affiliations:** 1 Department of Chemistry, Faculty of Science, King Saud University, P.O. Box 22452, Riyadh 11495, Saudi Arabia

**Keywords:** Chalcones, Fatty acid, Antioxidant activity, DPPH

## Abstract

**Background:**

Chalcone, an important intermediate of flavonoid synthetic pathway, has been shown to exhibit diverse biological and pharmacological activities such as anti- cancer, antioxidant, anti-inflammatory, etc.

**Results:**

In this study, a novel series of chalcones fatty acid esters **5b-e** and **6b-e** have been synthesized via the reaction of the respective chalcones with either palmitic or stearic acid. Another related class of compounds comprising 2,3-disubstituted chalcones **7b-d** and **8b(b’)-d** as well as 2-amino-6-(substituted-phenyl)-4-substitutedphenyl-nicotinonitrile derivatives **9a,c,e** have been also prepared by both electrophilic and Michael addition reactions, respectively, with the corresponding chalcones. The structures of all compounds are confirmed via a wide range of spectroscopic techniques including IR, ^1^H and ^13^C NMR, and mass spectra. Significantly, all synthesized compounds have been tested for their promising antioxidant activities via utilization of 1,1-biphenyl-2-picrylhydrazyl as a free radical scavenging reagent. Surprisingly, the results demonstrated that compound **5e** (68.58% at C = 2 μg/ml) was more effective as an antioxidant agent than the ascorbic acid, a commonly used antioxidant. Furthermore, the role and contribution of different functional groups on the antioxidant activity of the synthesized chalcone derivatives are also probed and rationalized in terms of their electronic and structural effect.

**Conclusion:**

Good activity was noted for chalcone fatty acid esters, with some members recorded higher antioxidant activity than ascorbic acid.

## Background

Naturally occurring chalcones as well as their synthetic analogues have been shown to exhibit interesting and diverse biological and pharmacological activities, including anticancer, antioxidant, anti-inflammatory, antimicrobial, and immunosupportive potential [1-4]. They are absorbed in the daily diet and appear to be promising cancer chemo-preventive agents. Generally, chalcones are synthesized by condensation reaction of aryl ketones and aromatic aldehydes, in the presence of suitable condensing agents.

Of particular relevance to the present work is the fact that the antioxidant properties of chalcones are highly influenced by the structure of the two aryl moieties in the backbone, i.e. the substitution on two aryl rings of chalcone molecule and their substitution patterns. In this context, hydroxyl substituent is proven to be one of the key groups that greatly enhance the antioxidant activity of chalcone as a result of its facile conversion to the corresponding phenoxy radicals through the hydrogen atom transfer mechanism [5].

In view of these findings, Torres de Pinedo *et al*. [6-8] have investigated the antioxidant activity of several phenolic derivatives and found that both dihydrocaffeoyl alcohol (3-(3,4-dihydroxyphenyl)-1-propanol) and galloyl alcohol (3,4,5-trihydroxybenzylic alcohol) are stronger antioxidants than hydroxyl tyrosol in their action as radical-scavengers and for protecting oil matrix against rancidity.

Owing to the strong demand for new antioxidant agents, it becomes very critical to explore novel scaffold for the design and synthesis of new antioxidant agents in order to help in the battle against pathogenic microorganisms.

Therefore, in this context, we describe facile approaches and procedures that enable the synthesis of some new chalcone fatty acid esters, incorporating fatty acid moiety directly attached to one of the phenolic positions. Furthermore, in order to understand the structural feature that makes a compound an effective lipophilic antioxidant, we have measured the radical-scavenging capacity of chalcone fatty acid esters having various acylation positions in the phenol ring along with the length and the nature of the fatty acid connected to the phenol ring. Finally, to gain more knowledge about the role of the double bond and the carbonyl group in the antioxidant activity, various addition and condensation reactions of the desired chalcones have been explored. Importantly, the antioxidant test used in this study evaluates the capacity of the synthesized compounds against scavenger 1,1-diphenyl-2-picrylhydrazyl (DPPH) radical species. DPPH is a stable radical that presents an absorption maximum at 516 nm [9-11].

## Results and discussion

Herein, we report the synthesis of novel series of fatty acid chalcone esters (**5b-e**) and (**6b-e**) by esterification reactions of some chalcone derivatives with fatty acids.

**Scheme 1:**
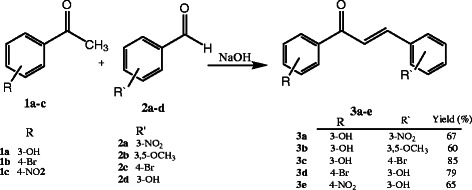
Preparation of chalcone derivatives.

Then, we prepared the chalcone precursors (**3a-e)** by the application of Claisen Schmidt condensation on selected acetophenones (**1a-c**) and benzaldehydes (**2a-d**) according to literature methods (Scheme 1) [5,11,12]. Details of the procedures and conditions are illustrated in experimental section.

**Scheme 2:**
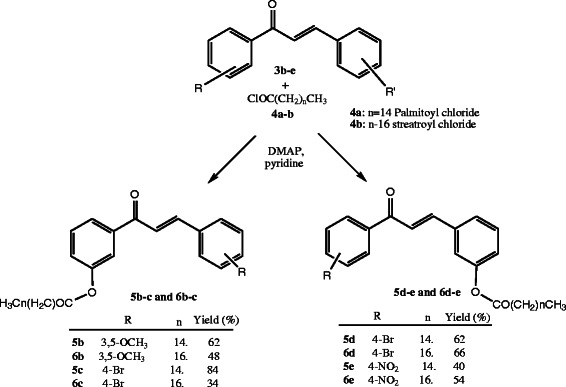
Preparation of compounds (5b-e) and (6b-e).

The synthesis of the desired fatty acid chalcone esters was then undertaken by esterification reaction of these freshly prepared chalcone derivatives with stearic or palmitic chlorides. The stearoyl/palmitoyl chlorides were prepared from stearic/palmitic acid and thionyl chloride (SOCl_2_) according to the literature procedures described in reference [11]. The stearoyl/palmitoyl chlorides were then reacted dropwise with the corresponding chalcones (**3b-e**) in pyridine, in the presence of dimethyl- amino-pyridine (DMAP) as a base [7,13,14] to give the desired (E)-3-(3-(substituted phenyl)acryloyl)phenyl/(E)-3-(3-(substituted phenyl)-3-oxoprop-1-en-1-yl)phenyl palmitate (**5b-e**) (E)-3-(3-(substituted phenyl)acryloyl)phenyl/(E)-3-(3-(substituted phenyl)-3-oxoprop-1-en-1-yl)phenyl stearate (**6b-e**) compounds, respectively, in very good yields, as illustrated in Scheme 2.

The structures of compounds (**5b-e)** and (**6b-e)** were verified by a combination of IR, and ^1^H and ^13^C NMR spectroscopy.

**Scheme 3:**
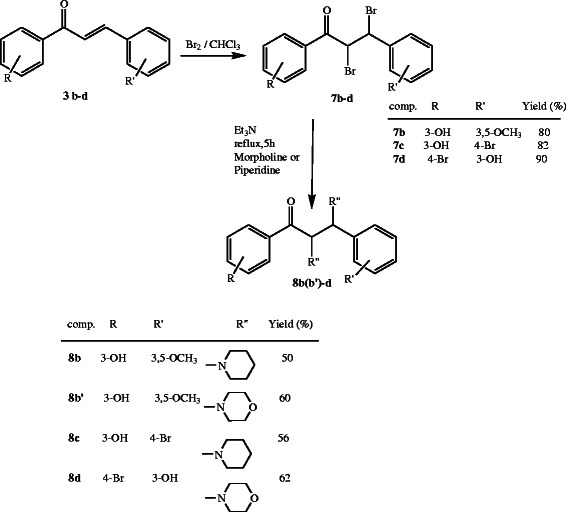
Preparation of 2,3-disubstituted chalcone derivatives.

The synthesis of the corresponding 2,3-dibromo chalcones (**7b-d)** was achieved by electrophilic addition reaction of the initially prepared chalcones (**3a-c**) and bromine in chloroform (Scheme 3). Similarly, the chemical structure of the produced compounds (**7b-d**) was identified and confirmed in view of their IR and NMR data.

Disubstitued chalcone derivatives comprising either piperidine or morpholine, namely 2,3-di(morpholin/piperidin-1-yl)substituted-1,3-diphenyl-propan-1-one (**8b(b’)-d)** were also successfully prepared by the nucleophilic substitution reaction of 2,3-dibromo chalcones (**7b-d**) by either piperidine or morpholine in absolute ethanol.

The desired products were isolated and purified by column chromatography. The synthetic procedures and reaction conditions for 2,3-di(morpholin/piperidin-1-yl)–substituted-1,3-diphenyl-propan-1-one **5a-c** are illustrated in Scheme 3. The spectral data (IR, ^1^H and ^13^C NMR) were used to identify the structure of these compounds.

**Scheme 4:**
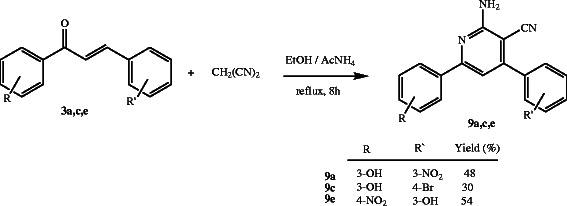
Preparation of compounds (9a,c,e).

Different synthetic approach has been used for the synthesis of the nicotinonitrile derivatives. In this regard, the synthesis of 2-amino-6-(substituted-phenyl)-4-substitutedphenyl nicotinonitrile (**9a,c,e**) was achieved by Michael addition reaction of selected chalcones **3a,c,e** with malonitrile in ethanol and in the presence of ammonium acetate (Scheme 4) [15]. The structures of these products were confirmed by their spectral data.

We note that all chalcones synthesis in this work are used in all reactions but for some of them no result was found. For example, **3a** give no reaction with fatty acid and **3a** and **3e** cannot react with bromine. These is can attribute to the effect of different substituents present in chalcone derivatives.

In this part, all the synthesized compounds were assessed for their antioxidant reactivity by monitoring spectrophotometirically the ability of the compounds to reduce 2,2-diphenylpicrylhydrazyl (DPPH), a commonly used radical scavenger.

**Figure 1:**
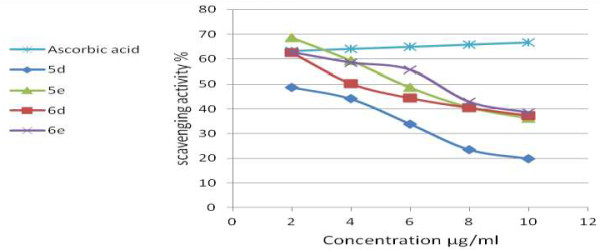
Antioxidant activity for 5d-e and 6d-e versus concentration.

A comparative assessment of these activities is shown in Table 1. The data (Table 1) clearly indicate that compounds having a meta ester group on B ring and/or para electron-withdrawing substituent as nitro group on A ring, as proposed for compounds (**5d-e and 6d-e**), gave very good activity. In particular, compounds **5e** and **6e** showed the strongest inhibition mainly at C = 2 μg/ml as illustrated in Figure 1. Importantly, the level of activity of these compounds is very close from the level of ascorbic acid particularly at low concentrations. The deceasing of the activities at higher concentrations could arise from the stearic bulk imparted by the long chain of the alkyl groups and by the large size of the DPPH.

**Table 1:** Antioxidants activities using (DPPH) radical scavenging method

**Radical-scavenging activity%**					**Comp.**
**2 μg/ml**	**4 μg/ml**	**6 μg/ml**	**8 μg/ml**	**10 μg/ml**	
63.10	64.00	64.86	65.81	66.70	ascorbic acid
39.65	44.03	48.85	52.00	56.00	3a
20.89	30.11	40.59	57.56	86.94	3b
43.67	34.78	27.62	19.83	17.81	3c
52.76	58.33	60.43	64.05	67.45	3d
25.26	32.25	33.67	33.51	36.79	3e
36.56	38.43	40.68	43.78	47.02	5b
29.01	32.23	36.43	39.12	51.24	5c
48.82	44.10	33.91	23.46	19.79	5d
68.59	59.33	48.66	40.62	36.23	5e
42.35	46.58	50.40	52.67	55.42	6b
31.18	38.06	45.55	52.29	54.15	6c
62.60	50.21	44.33	40.43	37.21	6d
62.71	58.53	55.62	42.84	38.52	6e
4.81	2.90	2.87	2.84	2.82	7b
6.44	5.58	5.01	4.17	3.68	7c
7.13	7.05	6.89	5.15	4.84	7d
42.87	38.43	35.35	35.16	32.72	8b
26.73	28.59	34.66	46.63	50.25	8b’
34.82	36.22	39.90	40.29	41.39	8c
33.97	30.65	29.23	28.93	24.60	8d
22.88	24.11	27.34	28.44	30.61	9a
12.06	12.12	12.15	15.17	21.91	9c
4.45	5.34	6.42	9.72	10.41	9e

**Figure 2:**
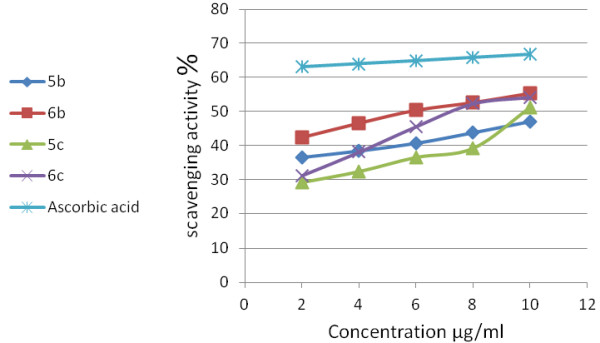
Antioxidant activity for 5b-c and 6b-c as a function of concentration.

In contrast, compounds (**5b** and **6b**), with a *meta* electron-withdrawing ester group on A, and *meta* donating methoxy group on B ring displayed moderate inhibition, but markedly less than ascorbic acid as shown in Figure 2. This behavior, however, is consistent with that reported by Murti *et al*. [9] on the synthesis and antioxidant activity of some chalcones and flavanoids.

Another important factor that affects the antioxidant activity of a compound is the nature of the fatty acid moiety. As can be seen in Figures 1 and 2, the presence of stearate ester attached to either ring A or B in the most commonly (excepted 5c at C = 2 μg/ml) synthesized chalcone derivatives has increased the antioxidant activity more than those containing palmitate ester. These results suggest that the substitution pattern of the hydroxyl group attached to ring A or B in the original chalcones may be crucial in enhancing their antioxidant reactivity [7,8,10].

In order to test the influence of double bond as well on the antioxidant activity of chalcone, we assessed the compounds obtained via addition reaction on these double bonds, i.e., compounds **7b-d** and **8b(b')-d**. Previous work has highlighted that electrophilic addition reaction in chalcone derivatives, having different substituents, can cause the antioxidant activity of these derivatives to decrease. Consistent with this finding, we noted that addition of bromine to chalcone compound **3c** in order to form compound **7c** has decreased its activity from 43.67% to 6.44% particularly, when the concentration (C) is 2 μg/ml. Interestingly, replacing the bromine by the piperidine as is the case in compound **8c** has resulted in marked increase in the antioxidant activity, leading to a maximum inhibition of 34.82% at C = 2 μg/ml. On the basis of the overall results, it can be concluded that the presence of double bond in chalcone structure is also important to improve the antioxidant activity [14,15].

Simliarly, we found that the presence of pyridine ring, cyano group and amino group in 2-amino-6-(substituted-phenyl)-4-substituted phenyl nicotinonitrile (compound **9c**) caused the inhibition to decrease dramatically compared to the parent chalcone **3e** i.e., from 43.67% to 12.06% [13,16-18].

**Figure 3:**
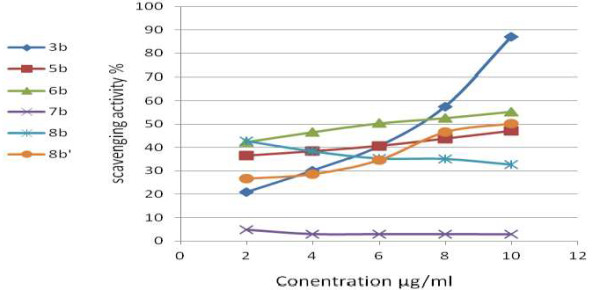
The effect of different reaction in the antioxidant activity.

**Figure 4:**
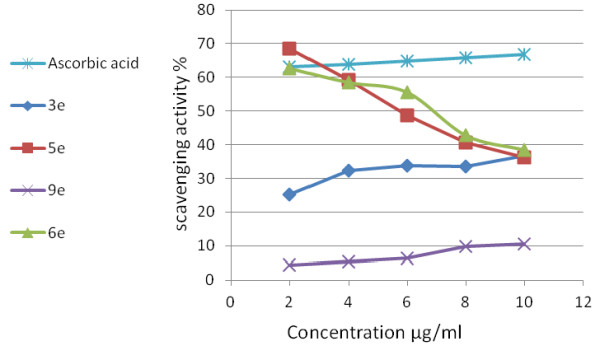
The effect of different reaction in the antioxidant activity.

Figures 3 and 4 summarize the net effect of the above mentioned modifications of the chalcone derivatives on the free radical scavenging activity of DPPH. As can be seen in Figure 3, for instance, the parent chalcone **3b**, has scavenging activity at C = 10 μg/ml about 86.94%. Addition of bromine to its double bond has caused its antioxidant activity to decrease to 2.82%, whereas the substitution by morpholine or piperidine resulted in an increase to 32.72% and 50.25%, respectively. On the other hand, the substitution of hydroxyl group by fatty acid in ring A or ring B had less effect on the antioxidant activity (47.02% and 55.42% respectively).

As for chalcone compound **3e**, the substitution of its hydroxyl group by the fatty acid to yield compound **5e** has increased its antioxidant activity, particularly at C = 2 μg/ml from 25.26% to 68.58%, a value that is higher than that of ascorbic acid. In the case of compound **6e,** an activity value (62.71%) close to that of ascorbic acid was obtained. However, upon condensation with malonitrile, (compound **9e**) this value has dramatically decreased to 21.9% as shown in Figure 4. The overall outcome of the antioxidant activity of this novel series of chalcone-based fatty acid esters revealed some new very active compounds that surpass the commonly used antioxidant such as ascorbic acid. Furthermore, this carefully probed structure-related activity studies added new insights and rationale to understand the role and contribution of different functional groups associated with chalcone.

## Conclusions

In this work, we have successfully synthesized a novel series of compounds comprising chalcone fatty acid esters, 2,3-disubstituted chalcones, and 2-amino-6-(substituted-phenyl)-4-substituted phenyl nicotinonitrile. The former class of compounds was prepared by esterification reactions of chalcones with fatty acid chlorides (streatoyl or palmitoyl) whilst the disubstitued analogues have been synthesized via electrophilic addition and Michael addition. The compounds were purified by recrystallization or column chromatography using appropriate solvent. The synthetic yields of the generated products ranged from 40 to 90% and their structures were established by spectral data (IR, NMR, MS). Finally, all of synthesized compound have been tested for their antioxidant activities using DPPH method. Good activity was noted for chalcone fatty acid esters, with some members recorded higher antioxidant activity than ascorbic acid.

### Experimental section

#### General

All chemicals used for the synthesis of the desired compounds were obtained from Merck and SD Fine chemicals. Melting points were determined using an electrothermal’s IA9000 series digital capillary melting point apparatus and used without correction. IR spectra were obtained, as KBr discs, a 1000-Perkin Elmer FT-IR spectrophotometer. Spectroscopic data were recorded as follows: ^1^H and ^13^C NMR spectra were acquired on a JEOL ECP-600 NMR in CDCl_3_ (or DMSO_d6_) using TMS as an internal standard. Chemical shifts are given in δ ppm. Mass spectra were collected using a direct inlet system (70 eV) with a VL detector (ES, 4000 V).

#### General procedure for the synthesis of 1,3-diphenyl-propenone derivatives 3a-e

A mixture of NaOH (22 g, 0.55 mol), acetophenone derivatives **1a-c** (0.43 mol), benzaldehyde derivatives **2a-d** (0.43 mol) was stirred at 25°C for 2–3 hours and left in the refrigerator overnight. The precipitated solid was then filtred off, washed with water and ethanol, dried, and crystallized from ethanol to yield yellow crystals.

#### *Synthesis of (E)-3-(3-(substitutedphenyl)acryloyl)phenyl or (E)-3-(3-(substitutedphenyl)-3-oxoprop-1-en-1-yl)phenyl palmitate stearate (5b-e) and (E)-3-(3-(substitutedphenyl)acryloyl)phenyl or (E)-3-(3-(substitutedphenyl)-3-oxoprop-1-en-1-yl)phenyl stearate* (*6b-e*)

To a suspension of fatty acid **4a-b** (0.08 mmol) in anhydrous CH_2_Cl_2_, the thionyl chloride (2.68 mmol) (0.12 ml) was added at 25°C under argon atmosphere and the mixture was stirred for 1 hour. One equivalent of the corresponding chalcone and DMAP in pyridine were then added and the mixture was stirred for 1 minute. The organic phase was extracted with water and diethyl ether, dried with sodium carbonate (Na_2_SO_4_), concentrated to dryness and purified by column chromatography (hexane–ethyl acetate (9:1) to give ***5b-e*** and ***6b-e***.

### (*E*)-3-(3-(3,5-dimethoxyphenyl)acryloyl)phenyl palmitate (5b)

Yellow powder, yield 62%, m.p. 116°C, m/z% 522 [M^+^] C_33_H_46_O_5_, IR (cm^-1^): 1702 (C = O) ester and 1654 (C = O) ketone, ^1^HNMR (CDCl_3_): 0.87 (t, 3H, -CH_3_), 1.24 (m, 24H, -CH_2_-), 1.62 (qu, 2H, -OCO-CH_2_-CH_2_-), 2.34 (t, 2H, -OCO-CH_2_), 3.83 (s, 6H, OCH_3_), 6.52 (t, 1H, H-4), 6.76 (d, 2H, H-2 & H-6), 7.1 (dd, 1H, H-4`), 7.37 (t, 1H, H-5`), 7.45 (d, 1H, *J* = 15.70 Hz, H-*α*), 7.55 (s, 1H, H-2`), 7.58 (d, 1H, H-6`), 7.72 (d, 1H, *J* = 15.70 Hz, H-*β*). ^13^CNMR (CDCl_3_): 13.2 (-CH_3_), 21.7 (-CH_2_-CH_3_), 23.7 (-CH_2_-CH_2_-CH_3_), 28.5 (-CH_2_-), 30.9(-CH_2_-CH_2_-COO-), 33.1 (-CH_2_-COO-), 54.5 (-OCH_3_), 102 (C-4), 105.5 (C-2 & 6), 114.2 (C-2`), 119.4 (C-6`),120.1 (C-4`), 121.5 (C-*α*), 129 (C-5`), 135.7 (C-1), 138.5 (C-1`), 144.4 (C-*β*), 155.4 (C-3`), 160.1 (C-3 & 5), 179.2 (-COO-), 189.7 (-C = O).

### (*E*)-3-(3-(4-bromophenyl)acryloyl)phenyl palmitate (5c)

Yellow powder, yield 84%, m.p. 126°C, m/z% 540 [M^+^] C_31_H_41_BrO_3_, IR (cm^-1^): 1702 (C = O) ester and 1654 (C = O) ketone, ^1^HNMR (CDCl_3_): 0.87 (t, 3H, -CH_3_), 1.24 (m, 24H, -CH_2_-), 1.62 (qu, 2H, -OCO-CH_2_-CH_2_-), 2.34 (t, 2H, -OCO-CH_2_), 7.08 (d, 1H, H-4`), 7.39 (t, 1H, H-5`), 7.52 (m, 7H, H-2, 6, 3, 5, *α*, 2`, 6`), 7.74 (d, 1H, *J* = 15.60 Hz, H-*β*). ^13^CNMR (CDCl_3_): 13.0 (-CH_3_), 21.6 (-CH_2_-CH_3_), 23.6 (-CH_2_-CH_2_-CH_3_), 28.4 (-CH_2_-), 30.6 (-CH_2_-CH_2_-COO-), 33.1 (-CH_2_-COO-), 113.8 (C-2`), 119.5 (C-*α*), 119.8 (C-6`), 121.2 (C-4`), 123.9 (C-4), 128.6 (C-2,6 & C-5`), 131.1 (C-3,5), 132.5 (C-1), 138.3 (C-1`), 142.4 (C-*β*), 155.4 (C-3`), 175.4 (-COO-), 189.3 (-C = O).

### (*E*)-3-(3-(4-bromophenyl)-3-oxoprop-1-en-1-yl)phenyl palmitate (5d)

Yellow powder, yield 62%, m.p. 132°C, m/z% 540 [M^+^] C_31_H_41_BrO_3_, IR (cm^-1^): 1704 (C = O) ester and 1652 (C = O) ketone, ^1^HNMR (CDCl_3_): 0.83 (t, 3H, -CH_3_), 1.24 (m, 28H, -CH_2_-), 1.59 (qu, 2H, -OCO-CH_2_-CH_2_-), 2.31 (t, 2H, -OCO-CH_2_), 6.89 (d, 1H, H-4), 7.11 (s, 1H, H-2), 7.18 (d, 1H, H-6), 7.26 (t, 1H, H-5), 7.41 (d, 1H, *J* = 15.70 Hz, H-*α*), 7.61 (d, 2H, *J* = 8.00 Hz, H-3`&5`), 7.73 (d, 1H, J = 15.70 Hz, H-*β*), 7.85 (2H, d, *J* = 8.00 Hz, H-2`& 6`). ^13^CNMR (CDCl_3_): 12.9 (-CH_3_), 21.5 (-CH_2_-CH_3_), 23.5 (-CH_2_-CH_2_-CH_3_), 28.5 (-CH_2_-), 30.7 (-CH_2_-CH_2_-COO-), 32.8 (-CH_2_-COO-), 113.8 (C-2), 116.9 (C-4), 120.2 (C-6), 120.6 (C-*α*), 128.9 (C-2`& 6`), 129.1 (C-5),130.8 (C3`& 5`), 134.9 (C-4`), 135.2 (C-1), 136.8 (C-1`), 144.1 (C-*β*), 155 (C-3), 178.4 (-COO-), 188.4 (-C = O).

### (*E*)-3-(3-(4-nitrophenyl)-3-oxoprop-1-en-1-yl)phenyl palmitate (5e)

Orange powder, yield 40%, m.p. 182°C, m/z% 507 [M^+^] C_31_H_41_NO_5_, IR (cm^-1^): 1701 (C = O) ester and 1653 (C = O) ketone, ^1^HNMR (CDCl_3_): 0.85 (t, 3H, -CH_3_), 1.22 (m, 24H, -CH_2_-), 1.46 (qu, 2H, -OCO-CH_2_-CH_2_-), 2.17 (t, 2H, -OCO-CH_2_), 6.90 (d, 1H, H-4), 7.26 (s, 1H, H-2), 7.27 (d, 1H, H-6), 7.33 (t, 1H, H-5), 7.70 (d, 1H, *J* = 15.50 Hz, H-*α*), 7.86 (d, 1H, *J* = 15.50 Hz, H-*β*), 8.36 (m, 4H, H-2`, 6`, 3`&5`). ^13^CNMR (CDCl_3_): 13.3 (-CH_3_), 21.4 (-CH_2_-CH_3_), 23.8 (-CH_2_-CH_2_-CH_3_), 28.1 (-CH_2_-), 30.6 (-CH_2_-CH_2_-COO-), 33.0 (-CH_2_-COO-), 114.5 (C-2), 118.1 (C-4), 119.8 (C-6), 121.2 (C-α), 123.2 (C-2`& 6`), 129.1 (C-5), 129.3 (C3`& 5`), 135 (C-1), 141.6 (C-1`), 145.3 (C-β), 149.2 (C-3), 157.4 (C-4`), 173.8 (-COO-), 187.9 (-C = O).

### (***E*)-3-(3-(3,5-dimethoxyphenyl)acryloyl)phenyl stearate (6b)**

Yellow powder, yield 48%, m.p. 114°C, m/z% 540 [M^+^] C_35_H_50_O_5_, IR (cm^-1^): 1705 (C = O) ester and 1654 (C = O) ketone, ^1^HNMR (CDCl_3_): 0.87 (t, 3H, -CH_3_), 1.24 (m, 28H, -CH_2_-), 1.62 (qu, 2H, -OCO-CH_2_-CH_2_-), 2.34 (t, 2H, -OCO-CH_2_), 3.83 (s, 6H, OCH_3_), 6.52 (t, 1H, H-4), 6.76 (d, 2H, H-2 & H-6), 7.1 (dd, 1H, H-4`), 7.38 (t, 1H, H-5`), 7.44 (d, 1H, *J* = 15.30 Hz, H-*α*), 7.55 (d, 1H, H-6`), 7.58 (s, 1H, H-2`), 7.72 (d, 1H, *J* = 15.3 Hz, H-*β*). ^13^CNMR (CDCl_3_): 13.1 (-CH_3_), 21.7 (-CH_2_-CH_3_), 23.7 (-CH_2_-CH_2_-CH_3_), 28.1 (-CH_2_-), 30.9(-CH_2_-CH_2_-COO-), 33.1 (-CH_2_-COO-), 54.5 (-OCH_3_), 101.9 (C-4), 105.4 (C-2 & 6), 114.1 (C-2`), 1119.2 (C-6`), 120.1 (C-4`), 121.4 (C-*α*), 128.9 (C-5`), 135.6 (C-1), 138.7 (C-1`), 144.2 (C-*β*), 155.1 (C-3`), 160 (C-3 & 5), 179.5 (-COO-), 189.6 (-C = O).

### (*E*)-3-(3-(4-bromophenyl)acryloyl)phenyl stearate (6c)

Yellow powder, yield 34%, m.p. 120°C, m/z% 568 [M^+^] C_33_H_45_BrO_3_, IR (cm^-1^): 1702 (C = O) ester and 1654 (C = O) ketone, ^1^HNMR (CDCl_3_): 0.87 (t, 3H, -CH_3_), 1.24 (m, 28H, -CH_2_-), 1.62 (qu, 2H, -OCO-CH_2_-CH_2_-), 2.34 (t, 2H, -OCO-CH_2_), 7.11 (d, 1H, H-4`), 7.37 (t, 1H, H-5`), 7.51 (m, 7H, H-2, 6, 3, 5, *α*, 2`, 6`), 7.74 (d, 1H, *J* = 15.70 Hz, H-*β*). ^13^CNMR (CDCl_3_): 13.0 (-CH_3_), 21.6 (-CH_2_-CH_3_), 23.6 (-CH_2_-CH_2_-CH_3_), 28.3 (-CH_2_-), 30.8 (-CH_2_-CH_2_-COO-), 33.1 (-CH_2_-COO-), 114.1 (C-2`), 119.5 (C-*α*), 119.9 (C-6`), 121.3 (C-4`), 123.9 (C-4), 128.8 (C-2,6 & C-5`), 131.1 (C-3,5), 132.6 (C-1), 138.3 (C-1`), 142.8 (C-*β*), 155.2 (C-3`), 175.4 (-COO-), 189.3 (-C = O).

### **(*E*)-3-(3-(4-bromophenyl)-3-oxoprop-1-en-1-yl)phenyl stearate** (**6d)**

Yellow powder, yield 66%, m.p. 136°C, m/z% 568 [M^+^] C_33_H_45_BrO_3_, IR (cm^-1^): 1704 (C = O) ester and 1652 (C = O) ketone, ^1^HNMR (CDCl_3_): 0.87 (t, 3H, -CH_3_), 1.24 (m, 28H, -CH_2_-), 1.62(qu, 2H, -OCO-CH_2_-CH_2_-), 2.34 (t, 2H, -OCO-CH_2_), 6.91 (d, 1H, H-4), 7.14 (s, 1H, H-2), 7.20 (d, 1H, H-6), 7.29 (t, 1H, H-5), 7.44 (d, 1H, *J* = 15.70 Hz, H-*α*), 7.64 (d, 2H, *J* = 8.40 Hz, H-3`& 5`), 7.76 (d, 1H, *J* = 15.70 Hz, H-*β*), 7.87 (2H, d, *J* = 8.40 Hz, H-2`& 6`). ^13^CNMR (CDCl_3_): 12.9 (-CH_3_), 21.5 (-CH_2_-CH_3_), 23.5 (-CH_2_-CH_2_-CH_3_) , 28.2 (-CH_2_-), 30.8 (-CH_2_-CH_2_-COO-), 32.8 (-CH_2_-COO-), 113.8 (C-2), 116.8 (C-4), 120.1 (C-6), 120.6 (C-*α*), 128.9 (C-2`& 6`), 129.1 (C-5), 130.8 (C3`& 5`), 135 (C-4`), 135.6 (C-1), 137 (C-1`), 144.1 (C-*β*), 155 (C-3), 178.4 (-COO-), 188.5 (-C = O).

### (*E*)-3-(3-(4-nitrophenyl)-3-oxoprop-1-en-1-yl)phenyl stearate (6e)

Yellow powder, , yield 54%, m.p. 184°C, m/z% 535 [M^+^] C_33_H_45_NO_5_, IR (cm^-1^): 1703 (C = O) ester and 1653 (C = O) ketone, ^1^HNMR (CDCl_3_): 0.87 (t, 3H, -CH_3_), 1.24 (m, 24H, -CH_2_-), 1.62 (q, 2H, -OCO-CH_2_-CH_2_-), 2.34 (t, 2H, -OCO-CH_2_), 6.90 (d, 1H, H-4), 7.25 (s, 1H, H-2), 7.29 (d, 1H, H-6), 7.31 (t, 1H, H-5), 7.70 (d, 1H, *J* = 15.50 Hz, H-*α*), 7.84 (d, 1H, *J* = 15.50 Hz, H-*β*), 8.36 (m, 4H, H-2`, 6`, 3` &5`).^13^CNMR (CDCl_3_): 13.2 (-CH_3_), 21.5 (-CH_2_-CH_3_), 23.6 (-CH_2_-CH_2_-CH_3_), 28.2 (-CH_2_-), 30.7 (-CH_2_-CH_2_-COO-), 32.8 (-CH_2_-COO-), 114.7 (C-2), 117.6 (C-4), 119.5 (C-6), 121 (C-α), 123.1 (C-2`& 6`), 128.8 (C-5), 129.2 (C3`& 5`), 135 (C-1), 141.7 (C-1`), 145.1 (C-β), 149.1 (C-3), 157.1 (C-4`), 173.7 (-COO-), 187.7 (-C = O).

### *Synthesis of 2,3-Dibromo-1,3-diphenyl-propan-1-one derivatives* (7b-d)

A mixture of chalcones derivatives **3b-d** (1 mmol) and bromine (2 ml) in chloroform (25 ml) was stirred for 1–4 h. After cooling, ethanol was added and the precipitated solid was collected by filtration. Recrystallization from ethanol afforded 2,3-dibromo-1,3-diphenyl-propan-1-one derivatives as white powder **7b-d**.

### *2,3-Dibromo-3-(3,5-dimethoxy-phenyl)-1-(3-hydroxy-phenyl)-propan-1-one* (7b)

White powder, yield 80%, m.p. 236°C, m/z% 442 [M^+^] C_17_H_16_Br_2_O_4_, IR (cm^-1^): 3410 (OH) and 1711 (C = O) ketone, ^1^HNMR (CDCl_3_): 3.95 (s, 6H, -OCH_3_), 6.18 (s, 1H, H-2), 6.34 (s, 1H, H-6), 6.56 (d, 1H, H-4), 6.76 (d, 1H, H-4'), 6.85 (d, 1H, *J* = 11.40 Hz, H-*β*), 6.86 (t, 1H, H-5'), 7.02 (d, 1H, *J* = 11.40 Hz, H-*α*), 7.78 (s, 2H, H-2' & H-6'). ^13^CNMR (CDCl_3_): 45.78 (C-*β*), 48.07 (C-*α*), 56.71 (-OCH_3_), 96.91 (C-4), 105.32 (C-2), 107.7 (C-6), 112.31 (C-2'), 122.08 (C-4'), 131.94 (C-6'), 135.9 (C-5'), 136.8 (C-1'), 138.32 (C-1), 150.04 (C-3'), 156.11 (C-3), 156.38 (C-5), 188.94 (C = O).

### 2**,3-Dibromo-3-(4-bromo-phenyl)-1-(3-hydroxy-phenyl)-propan-1-one (7c)**

White powder, yield 82%, m.p. >300°C, m/z% 460 [M^+^] C_15_H_11_Br_3_O_2_, IR (cm^-1^): 3461 (OH) and 1709 (C = O) ketone, ^1^HNMR (CDCl_3_): 5.56 (d,1H, *J* = 10.80 Hz, H-*β*), 6.01 (d, 1H, *J* = 10.80 Hz, H-*α*), 7.41 (d, 2H, *J* = 8.40 Hz, H-2 & H-6), 7.53 (d, 2H, *J* = 8.40 Hz, H-3 & H-5), 7.60 (d, 1H, H-4'), 7.72 (t, 1H, H-5'), 7.78 (s, 1H, H-2'), 7.81 (d, 1H, H-6').^13^CNMR (CDCl_3_): 46.95 (C-*β*), 51.96 (C-*α*), 114.42 (C-2'), 122.7 (C-4'), 129.92 (C-2& C-6), 131.7 (C-6'), 131.72 (C-3 & C-5), 132.3 (C-4), 135.8 (C-5'), 137.9 (C-1), 138.01 (C-1'), 151.43 (C-3'), 189.38 (C = O).

### *2,3-Dibromo-1-(4-bromo-phenyl)-3-(3-hydroxy-phenyl)-propan-1-one* (7d)

White powder, yield 90%, m.p. >300°C, m/z% 460 [M^+^] C_15_H_11_Br_3_O_2_, IR (cm^-1^): 3447 (OH) and 1683 (C = O) ketone, ^1^HNMR (CDCl_3_): 6.21 (d, 1H, H-4), 6.61 (s, 1H, H-2), 6.64 (d, 1H, *J* = 11.40 Hz, H-*β*), 6.67 (d, 1H, *J* = 11.40 Hz, H-*α*), 7,66 (t, 1H, H-5), 7.70 (d, 2H, *J* = 8.50 Hz, H-3' & H-5'), 7.84 (d, 1H, H-6), 7.93 (d, 2H, *J* = 8.50 Hz, H-2' & H-6'). ^13^CNMR (CDCl_3_): 42.4 (C-*β*), 46.85 (C-*α*), 111.1 (C-4), 112.5 (C-2), 114.95 (C-6),117.4 (C-4'), 130.36 (C-2' & C-6'), 132.45 (C-3' & C-5'), 132.78 (C-5), 135.5 (C-1'), 137.01 (C-1), 149.84 (C-3), 189.26 (C = O).

### *Synthesis of 2,3-Disubstituted-1,3-diphenyl-propan-1-one derivatives* (8b(b’)-d)

A mixture of 2,3-dibromo-1,3-diphenyl-propan-1-one derivatives **7a-c** (0.01 mol) and morpholine or pepiridine (0.01 mol) in ethanol (10 ml) was heated in oil-bath for 5 h. After cooling, 50 ml of water was added and the organic phase was extracted, then washed with 10% of hydrochloric acid (HCl) (3 × 50 ml), dried with sodium carbonate (Na_2_SO_4_) and concentrated to dryness. The isolated solid was then purified by column chromatography on silica gel using ethyl acetate/hexane (3:7) as eluent to give 2,3-disubstituted-1,3-diphenyl-propan-1-one derivatives **8a-d** as yellow powders.

### *3-(3,5-Dimethoxy-phenyl)-1-(3-hydroxy-phenyl)-2,3-di-piperidin-1-yl-propan-1-one* (8b)

Yellow-orange powder, yield 50%, m.p. 225°C, m/z% 452 [M^+^] C_27_H_36_N_2_O_4_, IR (cm^-1^): 3427 (OH) and 1634 (C = O) ketone, ^1^HNMR (CDCl_3_): 1.25 (s, 12H, H-3-5 for piperidine), 1.66 (t, 8H, H-2 and H-6 for piperidine), 3.13 (d, 1H, H-*β*), 3.52 (d, 1H, H-*α*), 3.93 (s, 6H, -OCH_3_), 5.27 (s, 2H, H-2 & H-6), 5.52 (s, 1H, H-4), 6.58 (s, 2H, H-4' & H-5'), 7.62 (s, 2H, H-2' & H-6'). ^13^CNMR (CDCl_3_): 24.1 (C-3 for piperidine), 25.1 (C-5 for piperidine), 26.2 (C-4 for piperidine), 37.1 (C- *β*), 44.6 (C-*α*), 47.5 (C-6 for piperidine), 48.2 (C-2 for piperidine), 56.5 (-OCH_3_), 101.9 (C-4),108.2 (C-2), 108.3 (C-6), 109.6 (C-2'), 121 (C-4'), 134.8 (C-5' & C-6'), 139.4 (C-1'), 146 (C-1), 148.3 (C-3'), 156.3(C-3 & C-5), 187.8 (C = O).

### *3-(3,5-Dimethoxy-phenyl)-1-(3-hydroxy-phenyl)-2,3-di-morpholin-4-yl-propan-1-one* (8b’)

Yellow powder, yield 60%, m.p. 253°C, m/z% 456 [M^+^] C_25_H_32_N_2_O_6_, IR (cm^-1^): 3424 (OH) and 1642 (C = O) ketone, ^1^HNMR (CDCl_3_): 3.01 (s, 8H, H-2 and H-6 for morpholine) , 3.41 (d, 1H, H-*β*), 3.58 (d, 1H, H-*α*), 3.77 (s, 8H, H-3 and H-5 for morpholine), 3.97 (s, 6H, -OCH_3_), 5.41 (s, 2H, H-2 & H-6), 5.59 (s, 1H, H-4), 6.62 (s, 2H, H-4' & H-5'), 7.53 (s, 2H, H-2' & H-6').^13^CNMR (CDCl_3_): 40.6 (C-*β*), 44,5 (C-2 and C-6 for morpholine), 45.8 (C-*α*), 56.6 (-OCH_3_), 65.5 (C-3 and C-5 for morpholine), 96.9 (C-4), 101.8 (C-2), 101.9 (C-6), 111.2 (C-2'), 114.3 (C-4'), 134.3 (C-5' & C-6'), 138.9 (C-1'), 144.7 (C-1), 157 (C-3'), 160.9 (C-3& C-5), 188.1 (C = O).

### ***3-(4-Bromo-phenyl)-1-(3-hydroxy-phenyl)-2,3-di-piperidin-1-yl-propan-1-one*** (**8c)**

Yellow powder, yield 56%, m.p. 220°C, m/z% 470 [M^+^] C_25_H_31_BrN_2_O_2_, IR (cm^-1^): 3447 (OH) and 1656 (C = O) ketone, ^1^HNMR (CDCl_3_): 1.25 (s, 12H, H-2, H-3, H-5 and H-6 for piperidine), 1.73 (t, 8H, H-2 and H-6 for piperidine), 4.37 (s, 1H, H-*β*), 4.38 (s, 1H, H-*α*), 7.47 (d, 2H, *J* = 8.40 Hz, H-2 & H-6), 7.51 (d, 2H, *J* = 8.40 Hz, H-3 & H-5), 7.49 (d, 1H, H-4'), 7.67 (t, 1H, H-5'), 7.74 (s, 1H, H-2'), 7.85 (d, 1H, H-6'). ^13^CNMR (CDCl_3_): 24.3 (C-3 for piperidine), 25.4 (C-5 for piperidine), 26.4 (C-4 for piperidine), 37.1 (C- *β*), 44.6 (C-*α*), 47.1 (C-6 for piperidine), 47.6 (C-2 for piperidine), 118.1 (C-2'), 122.9 (C-4'), 131.4 (C-2& C-6), 131.5 (C-3& C-5), 131.9 (C-6'), 132.2 (C-4), 134.1 (C-5'), 135.1 (C-1), 135.2 (C-1'), 149.3 (C-3'), 193.7 (C = O).

### *1-(4-Bromo-phenyl)-3-(3-hydroxy-phenyl)-2,3-di-morpholin-4-yl-propan-1-one* (8d)

Yellow-orange powder, yield 62%, m.p. 245°C, m/z% 574 [M^+^] C_23_H_27_BrN_2_O_4_, IR (cm^-1^): 3442 (OH) and 1651 (C = O) ketone, ^1^HNMR (CDCl_3_): δ 2.59 (s, 8H, H-2 and H-6 for morpholine), 3.77 (s, 8H, H-3 and H-5 for morpholine), 4.27 (s, 1H, H-*β*), 4.47 (s, 1H, H-*α*), 6.05 (s, 2H, H-2 & H-4), 7.51 (d, 2H, *J* = 8.50 Hz, H-3' & H-5'), 7.59 (t, 1H, H-5), 7.70 (d, 2H, *J* = 8.50 Hz, H-2' & H-6'), 7.72 (d, 1H, H-6) ppm. ^13^CNMR (CDCl_3_): δ 40.4 (C-*β*), 43.6 (C-2 and C-6 for morpholine), 46.6 (C-*α*), 64.6 (C-3 and C-5 for morpholine), 111.4 (C-2 & C-4), 111.8 (C-6), 112.1 (C-4'), 129.1 (C-2'& C-6'), 131.2 (C-3' & C-5'), 134.8 (C-5), 137.8 (C-1'), 139.6 (C-1), 159.5 (C-3), 185.1 (C = O) ppm.

### *Synthesis of 2-amino-4,6-diphenyl-nicotinonitrile derivatives* (9a,c,e)

A mixture of chalcones **3a, c** and **f** (10 mmol), malononitrile (10 mmol) and ammonium acetate (80 mmol) in ethanol (50 ml) was heated under reflux for 5 h. The solid product was filtered off, washed with ethanol, dried and recrystallized from ethanol to yield 2-amino-4,6-diphenyl-nicotinonitrile derivatives **9a-c.**

### *2-Amino-6-(3-hydroxy-phenyl)-4-(3-nitro-phenyl)-nicotinonitrile* (9a)

Brown powder, yield 48%, m.p. 286°C, m/z% 332 [M^+^] C_18_H_12_N_4_O_3_, IR (cm^-1^): 3440 (OH), 3253, 3357 (NH_2_), 2216 (CN), 1653 (C = N), ^1^HNMR (CDCl_3_): δ 6.96 (m, 4H, H-5, H-2', H-4', H-6'), 7.07 (s, 2H, NH_2_), 7.32 (t, 1H, H-5'), 7.83 (t, 1H, H-5"), 8.11 (d, 1H, H-6"), 8.36 (d, 1H, H-4"), 8.46 (s. 1H, H-2"), 9.79 (s, 1H, -OH) ppm. ^13^CNMR (CDCl_3_): δ 93.9 (C-3), 114.5 (C-4'), 114.9 (C-5), 115.7 (C-2'), 117.7 (CN),118.4 (C-6'), 122.6 (C-2"), 123.3 (C-4"), 129 (C-5"), 129.5 (C-5'), 134.5 (C-6"), 137.7 (C-1"), 138 (C-1'), 147 (C-3"), 149.4 (C-4), 153.2 (C-3'), 156.6 (C-6), 171.7 (C-2) ppm.

### *2-Amino-4-(4-bromo-phenyl)-6-(3-hydroxy-phenyl)-nicotinonitrile* (9c)

Yellow-green powder, yield (30%), m.p. 250°C, m/z% 395 [M^+^] C_18_H_12_BrN_3_O, IR (cm^-1^): 3431 (OH), 3226, 3335 (NH_2_), 2216 (CN), 1653 (C = N), ^1^H NMR (CDCl_3_): δ 6.88 (d, 1H, H-6'), 7.05 (s, 2H, NH_2_), 7,18 (s, 1H, H-2'), 7.28 (t, 1H, H-5'), 7.51 (s, 1H, H-5), 7.53 (d, 1H, H-4'), 7.64 (d, 2H, *J* = 9.00 Hz, H-2" & 6"), 7.77 (d, 2H, *J* = 9.00 Hz, H-3"& 5"), 9.63 (s, 1H, OH) ppm. ^13^CNMR (CDCl_3_): δ 85.8 (C-3), 108.7 (C-2'), 113.6 (C-5), 116.4 (CN),116.7 (C-4'), 117.7 (C-6'), 122.7 (C-4"), 129.1 (C-5'), 130.1 (C-2"&C-6"), 131.3 (C-3"&C-5"), 135.4 (C-1"), 138.4 (C-1'), 152.9 (C-4), 157.2 (C-3'), 158.5 (C-6), 160.3 (C-2) ppm.

### *2-Amino-4-(3-hydroxy-phenyl)-6-(4-nitro-phenyl)-nicotinonitrile* (9e)

Brown powder, yield 54%, m.p. 230°C, m/z% 332 [M^+^] C_18_H_12_N_4_O_3_, IR (cm^-1^): 3388 (OH), 3242, 3388 (NH_2_), 2218 (CN), 1635 (C = N), ^1^H NMR (CDCl_3_): δ 6.82 (s, 1H, H-2"), 6.97 (m, 3H, H-4", H-6", H-5), 7.04 (s, 2H, NH_2_), 7.32 (t, 1H, H-5"), 8.35 (m, 4H, H-2', 6' & H-3', H 5'), 9.81 (s, 1H, OH) ppm. ^13^CNMR (CDCl_3_): δ 98.2 (C-3), 114.6 (C-5), 115.1 (C-2"), 115.9 (C-4"), 117.6 (CN),118.6 (C-6"), 123.1 (C-3'&C-5'), 129.2 (C-5"), 129.6 (C-2'&C-6'), 137.9 (C-1"), 143.1 (C-1'), 147.1 (C-4'), 153.4 (C-4), 156.3 (C-3'), 158.3 (C-6), 163.5(C-2) ppm.

### Preparation of different simples to assess the antioxidant activity

Stock solution of free radical scavenger 2,2-diphenylpicrylhydrazyl (DPPH) 100 μL was added to 3.0 ml of methanol and the absorbance was recorded at 516 nm by UV–VIS spectrophotometer. Different concentrations of the synthesized compounds (2, 4, 6, 8, and 10 μ g/ml) were prepared also in methanol. All sample solutions, 1.0 ml each, is diluted with 3.0 ml methanol and 100 μL DPPH was added. The test tubes were kept for 30 min in the dark to complete the reaction. The absorbance of each test tube was recorded at 517 nm on UV–VIS spectrophotometer against methanol as a blank. The DPPH free radical scavenging activity was calculated using the following formula:

$$\begin{array}{l} \text{Scavenging}\;\text{activity}\;\left(\%\right)=\frac{\left[\text{Absorbance}\;\text{of}\;\text{control}-\text{Absorbance}\;\text{of}\;\text{test}\;\text{sample}\right]}{\text{Absorbance}\;\text{of}\;\text{control}}\\ \;\times 100 \end{array}$$

Where: control is absorbance of a DPPH solution without compound and ascorbic acid was used as the free radical scavenger reference compound.

## Competing interests

The authors declared that they have no competing interests.

## Authors’ contributions

L, AK, and A carried out the acquisition of data, analysis and interpretation of data collected and involved in drafting of manuscript, revision of draft for important intellectual content and give final approval of the version to be published. All authors read and approved the final manuscript.
